# A prebiotic diet modulates the oral microbiome composition and results in the attenuation of oropharyngeal candidiasis in mice

**DOI:** 10.1128/spectrum.01734-23

**Published:** 2023-09-06

**Authors:** Roberto Vazquez-Munoz, Angela Thompson, Takanori Sobue, Anna Dongari-Bagtzoglou

**Affiliations:** 1 Department of General Dentistry, The University of Connecticut Health Center, Farmington, Connecticut, USA; Uniwersytet Medyczny w Bialymstoku, Bialystok, Poland

**Keywords:** prebiotic, *Candida albicans*, candidiasis, oral microbiome, *Lactobacillus*, fungal infection, dysbiosis, xylooligosaccharides

## Abstract

Oral bacteria can influence the ability of *Candida albicans* to cause oropharyngeal candidiasis (OPC). We recently reported that a *Lactobacillus johnsonii*-enriched oral microbiota reduced *C. albicans* virulence in an immunosuppressed OPC mouse model. As a follow-up, in this work, we aimed to enrich the resident oral *Lactobacillus* communities with a prebiotic diet to further assess their effect on the severity of OPC. We tested the effect of a prebiotic xylo-oligosaccharides (XOS)-enriched diet in the oral global bacterial composition and severity of OPC. We assessed changes in the oral microbiome composition via 16S-rRNA gene high-throughput sequencing, validated by qPCR. The impact of the prebiotic diet on *Candida* infection was assessed by quantifying changes in oral fungal and bacterial biomass and scoring tongue lesions. Contrary to expectations, oral *Lactobacillus* communities were not enriched by the XOS-supplemented diet. Yet, XOS modulated the oral microbiome composition, increasing *Bifidobacterium* abundance and reducing enterococci and staphylococci. In the OPC model, the XOS diet attenuated *Candida* virulence and bacterial dysbiosis, increasing lactobacilli and reducing enterococci on the oral mucosa. We conclude that XOS attenuates *Candida* virulence by promoting a bacterial microbiome structure more resilient to *Candida* infection.

**IMPORTANCE:**

This is the first study on the effects of a prebiotic diet on the oral mucosal bacterial microbiome and an oropharyngeal candidiasis (OPC) mouse model. We found that xylo-oligosaccharides change the oral bacterial community composition and attenuate OPC. Our results contribute to the understanding of the impact of the oral bacterial communities on *Candida* virulence.

## INTRODUCTION

Oropharyngeal candidiasis (OPC) is the most prevalent oral fungal infection in individuals with weakened immune systems (1). The main etiological cause of OPC is *Candida albicans,* a fungal member of the healthy mucosal microbiota (2, 3). *C. albicans* infection can induce oral mucosal dysbiosis, decreasing the microbiome diversity due to a bloom of disease-associated bacterial species (4). In mouse oral infection models and certain human infections, *Candida*-driven dysbiosis favors the growth of enterococci and streptococci that, in turn, may increase fungal virulence (5 – 8).

*Candida* infections, such as OPC, are difficult to treat due to the rise of multidrug-resistant *C. albicans* strains (9) and the limited availability of antifungals; thus, there is a growing interest in alternative microbiome-based therapeutic strategies. In this regard, interest in probiotic bacteria with antifungal activity is growing (10). For example, certain species of lactobacilli have antifungal properties against *C. albicans* and reduce the severity of candidiasis in murine intestinal infection models (11, 12). We recently found that a *Lactobacillus johnsonii*-enriched oral microbiota is associated with reduced *C. albicans* virulence in a cortisone-immunosuppressed mouse oral infection model (13). We isolated, sequenced the genome (14), and phenotypically characterized a novel *L. johnsonii* strain (strain MT4) from the oral cavity of these mice. Strain MT4 displayed significant anticandidal properties in planktonic and biofilm growth models *in vitro*. We also identified key genetic and phenotypic traits that may be associated with this activity (15).

As a follow-up, in this work, we tested a prebiotic diet to enrich the oral *Lactobacillus* communities in cortisone-immunosuppressed mice in order to further assess their effect on the severity of OPC. Prebiotics are non-digestible food supplements that selectively favor the growth of probiotic bacteria (16). We used dietary xylo-oligosaccharides (XOS), oligomers formed by β-1,4 linked xylose monomers, to increase oral lactobacilli selectively. XOS-supplemented diets have been associated with an increase in *Lactobacillus* species and other health-associated bacteria such as bifidobacteria, and a decrease in disease-associated bacteria, such as enterococci and clostridia, in the gut (17, 18).

This study is the first investigation of the effects of a prebiotic diet on the oral mucosal bacterial microbiome in mice. We demonstrated that an XOS-based prebiotic diet attenuates *Candida* virulence in a mouse OPC model and partially restores the mucosal bacterial dysbiosis associated with this infection, by increasing lactobacilli and reducing enterococci.

## RESULTS

### Dietary XOS induces changes in the oral bacterial microbiome composition

**Fig 1:**
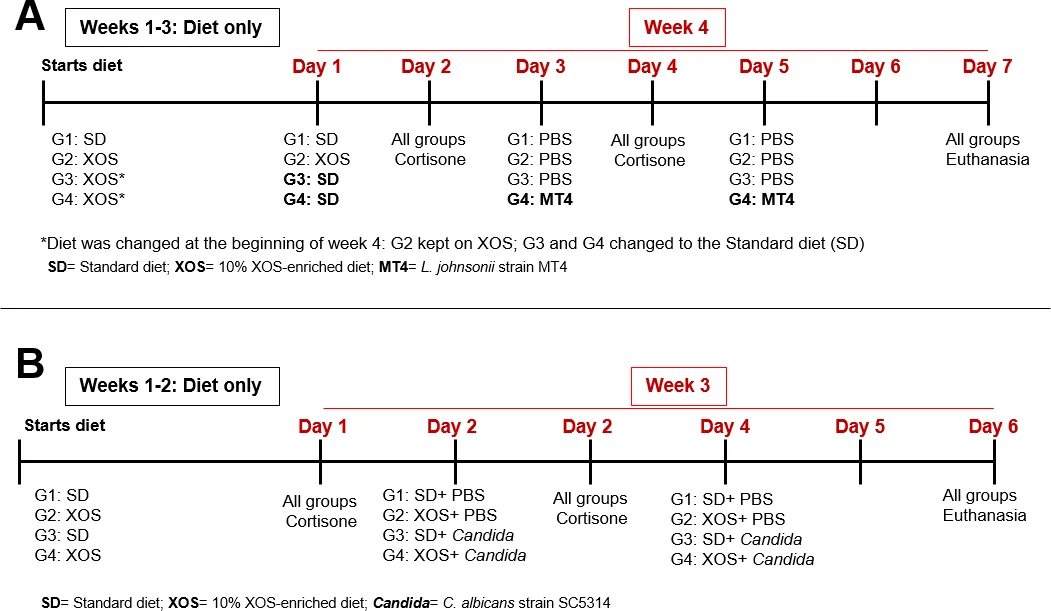
Experimental design. (**A**) Impact of XOS diet and MT4 reconstitution on oral bacterial communities. (**B**) Effect of XOS diet on *C. albicans* infection.

**Fig 2:**
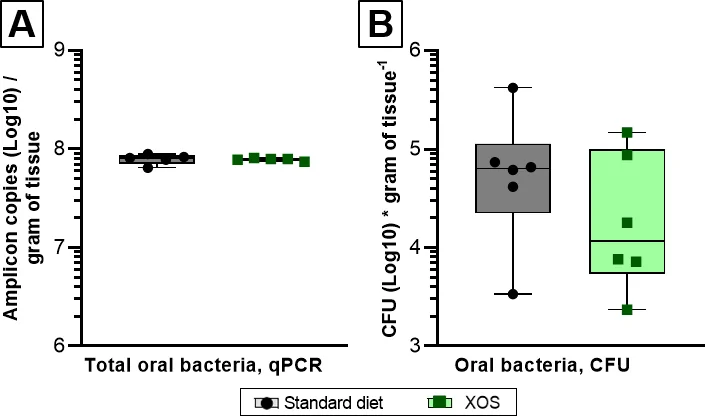
Effect of XOS consumption on the total oral bacterial biomass, as assessed by (**A**) qPCR (16S rRNA amplicons) and (**B**) CFU counts. Unpaired *t*-test.

**Fig 3:**
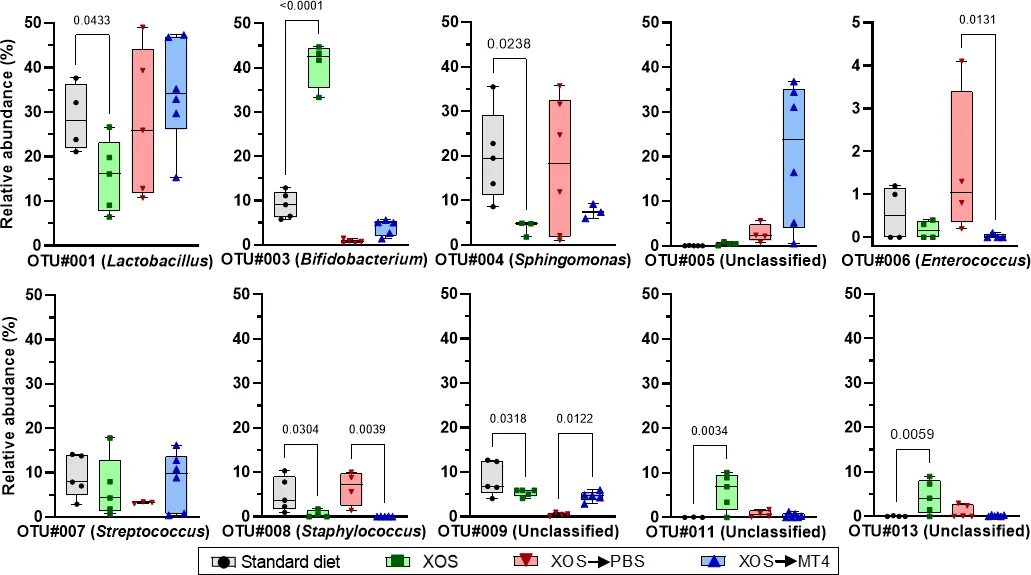
Effect of XOS diet and MT4 reconstitution post-XOS diet on the relative abundance of the most abundant OTUs. One-way ANOVA with uncorrected Dunn’s test.

To assess global changes in the oral bacterial composition in response to an XOS-supplemented diet, mice received the prebiotic for 3 weeks (Fig. 1A-experimental design). Mice were kept on the 10% XOS diet for 3 weeks because previous studies had shown that this duration is effective in modifying gut microbiome composition in rodents (17, 19). When compared to the standard diet, the XOS diet had no effect on body weight or food consumption (Fig. S1A and B). The XOS diet had no significant impact on the oral total bacterial biomass, as assessed by 16S rRNA gene copy numbers and viable counts on non-selective media (Fig. 2). High-throughput 16S rRNA gene V4 sequence analyses revealed that, contrary to expectations, the relative abundance of lactobacilli was significantly reduced by XOS, while bifidobacteria were significantly increased [Fig. 3, operational taxonomic unit (OTU)#001 and OTU#003, respectively]. These changes were confirmed by genus-specific qPCR on tongue tissues (Fig. S2A and B). The effect of XOS on *Lactobacillus* growth was also confirmed by *in vitro* cultures of bacteria recovered from oral swabs. Lactobacilli showed reduced growth in man, rogosa, and sharpe (MRS) supplemented with XOS, compared to MRS with dextrose (Fig. S3). Similar to the oral cavity, the XOS diet significantly increased fecal bifidobacteria but had no significant effect on gut lactobacilli (Fig. S4). A significant reduction in the relative abundance of oral *Sphingomonas* (OTU#004), *Staphylococcus* (OTU#008)*,* and OTU#009 (unclassified) was noted in the XOS group, whereas OTU#011 and OTU#013 were significantly increased. Consistent with our previous findings (13), mean relative abundance data in the standard diet group indicated that *Lactobacillus* was represented by the most abundant OTU (28.7% ± 6.5%), followed by *Sphingomonas* (20% ± 9.1%), *Streptococcus* (9.1% ± 4.3%), and *Bifidobacterium* (9.1% ± 2.7%). In the XOS group, *Bifidobacterium* was by far the most abundant OTU (40.8% ± 4.4%), followed by *Lactobacillus* (15.6% ± 7.3%) (Fig. 3; Table S1).

**Fig 4:**
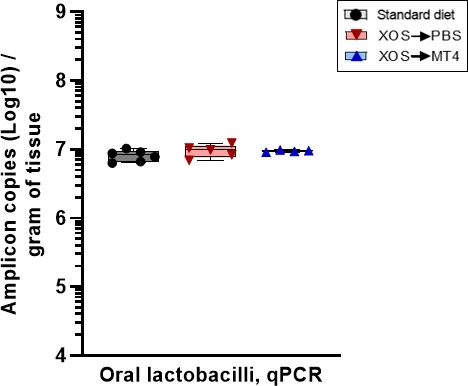
Resident oral lactobacilli populations rebound within a week once the XOS diet is discontinued.

**Fig 5:**
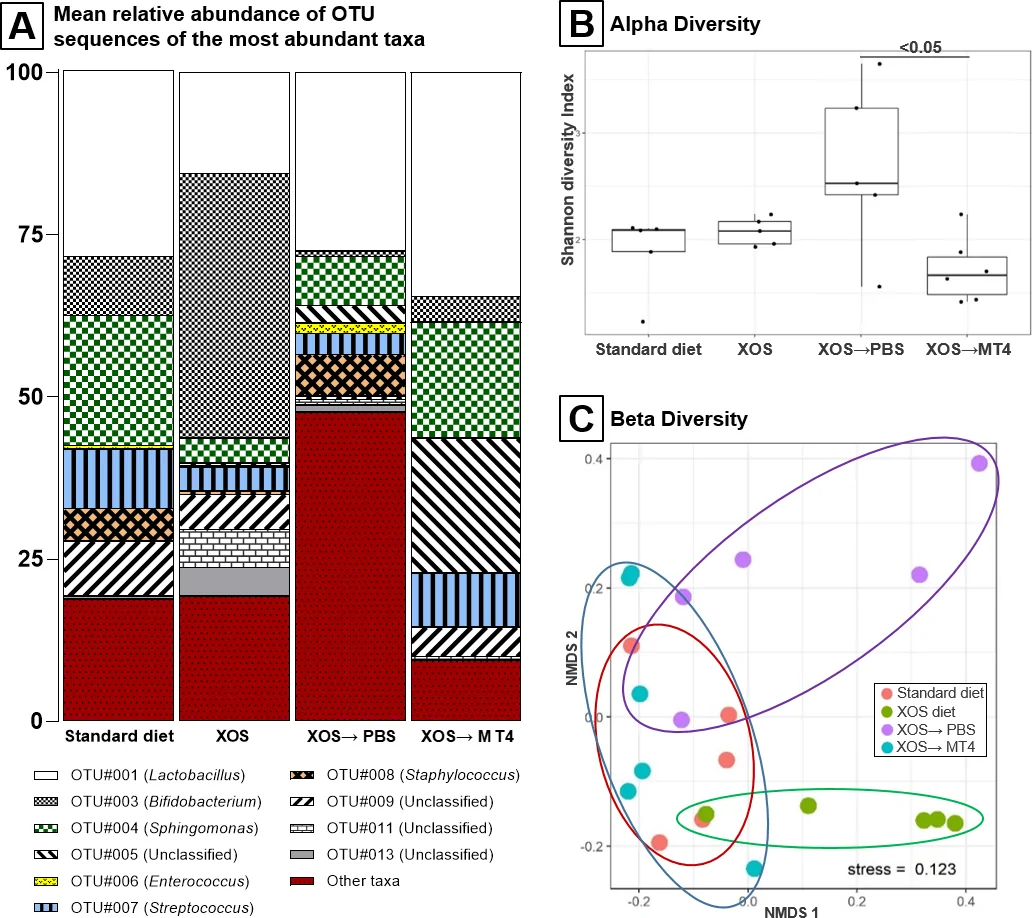
MT4 reconstitution reversed the XOS-induced changes in the oral bacterial microbiome. (**A**) Mean relative abundance of OTU sequences of the most abundant oral taxa in the four groups. (**B**) Alpha diversity as assessed by Shannon Index. (**C**) Beta diversity, as assessed by non-metric multidimensional scaling (NMDS) ordination based on Bray-Curtis dissimilarities of community structure. Permutational multivariate analyses of variance for Adonis, *P* < 0.01.

The reduction in the relative and absolute abundance of oral lactobacilli by the XOS diet raised the question of whether these communities could be restored by orally inoculating mice with strain MT4 after the XOS diet was discontinued. In this experimental design, two groups of mice were inoculated twice with MT4 or PBS (control), within the course of a week (groups 3 and 4, Fig. 1A). MT4 inoculation restored the *Lactobacillus* communities; however, there was no significant difference with the PBS control group, showing that resident lactobacilli rebound within a week once the XOS diet is discontinued (Fig. 3 and 4). Interestingly, MT4 supplementation partially restored the oral microbiome structure and the relative abundance of OTUs other than lactobacilli (Fig. 5A; Table S1). In particular, the relative abundance of *Bifidobacterium, Sphingomonas,* and *Staphylococcus* species in the MT4-inoculated group resembled those of the standard diet group. In the MT4-inoculated group, there was a significant reduction in enterococci compared to the PBS control group (Fig. 3 and 5A OTU#006; Table S1) which was confirmed by qPCR (Fig. S5). Alpha diversity analyses, as expressed by the Shannon index, showed significant variability in mice of the PBS control group, whereas the MT4-supplemented group was more homogeneous and had a significantly lower alpha diversity (Fig. 5B). Beta diversity analyses confirmed that the standard diet and MT4-supplemented groups exhibited similar oral bacterial community structures, which differed significantly from the PBS control group (Fig. 5C). Together these results show that the introduction of strain MT4 at least partially restores a diet-perturbed oral microbiome in mice.

### XOS diet reduced the severity of OPC and the impact of *Candida* on the oral bacterial microbiome

**Fig 6:**
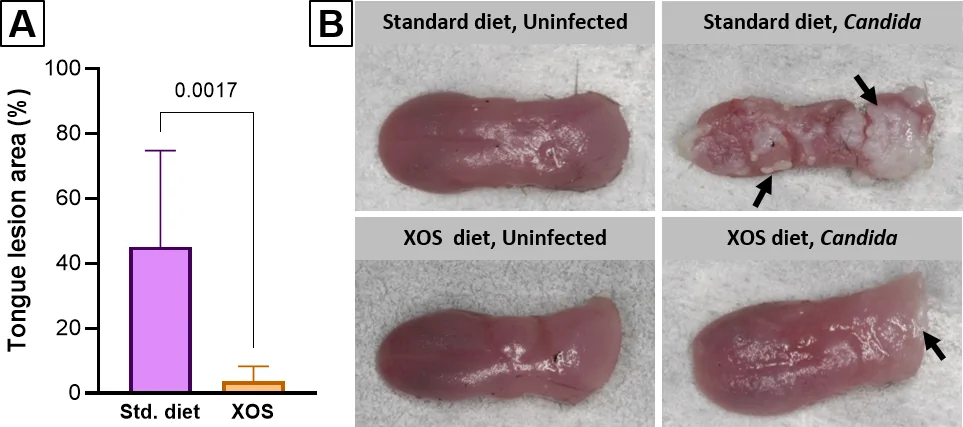
Effect of XOS diet on *Candida* virulence. (**A**) Mean tongue biofilm surface area in mice receiving standard or XOS diet (Unpaired *t*-test). (**B**) Representative images of infected and uninfected mouse tongues on standard or XOS diet. Black arrows point to biofilms.

**Fig 7:**
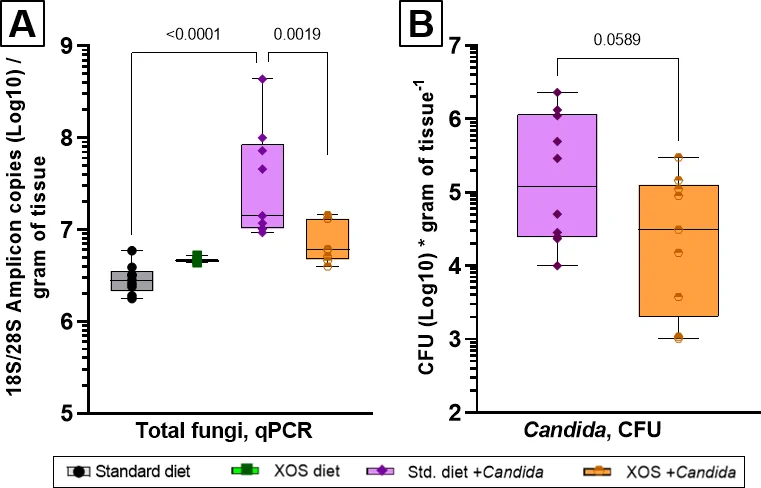
Effect of XOS diet on the oral fungal biomass compared to the standard diet. (**A**) qPCR (18S/28S rRNA intergenic region amplification, one-way ANOVA with the uncorrected Dunn’s test) and (**B**) CFU counts. Unpaired *t*-test.

We noticed that the XOS diet reduced the collective relative abundance of bacterial taxa with possible synergy in *C. albicans* pathogenesis (5 – 8)(20). Specifically, *Enterococcus*, *Streptococcus*, and *Staphylococcus*, which represented collectively 14.7% of the OTUs in the standard diet group, represented only 4.4% in the XOS diet group (Table S1). This raised the possibility that a reduction in synergistic bacteria may reduce susceptibility to OPC. Thus, during the third week of the XOS or standard diet, cortisone-immunosuppressed mice were infected with *C. albicans* in order to assess virulence (Fig. 1B, experimental design). Macroscopic examination of the tongues showed that the XOS diet was associated with a reduction in the incidence and severity of tongue lesions in *Candida*-infected mice. While all mice on the standard diet developed biofilm lesions on the dorsal surface (average biofilm surface area 49.8% ± 25.7%) most infected mice on the XOS diet showed smaller lesions or no lesions at all (average 3.6% ± 4.3% biofilm surface area) (Fig. 6A and B). These results were consistent with the significantly lower fungal biomass in the XOS diet compared to the standard diet, as assessed by qPCR and viable counts on antibiotic-supplemented SDA agar (Fig. 7A and B). Weight loss tended to be lower in infected mice on the XOS diet compared to the standard diet although food consumption did not differ, suggesting that possible systemic effects of oral infection were not completely reversed by the prebiotic diet (Fig. S1A and B). Furthermore, 18S qPCR revealed that the XOS diet alone did not affect indigenous fungal communities in uninfected mice (Fig. 7A), and XOS-supplemented yeast, peptone, and dextrose (YPD) did not affect *C. albicans* growth *in vitro* (Fig. S6), indicating that this prebiotic does not have direct anticandidal properties.

**Fig 8:**
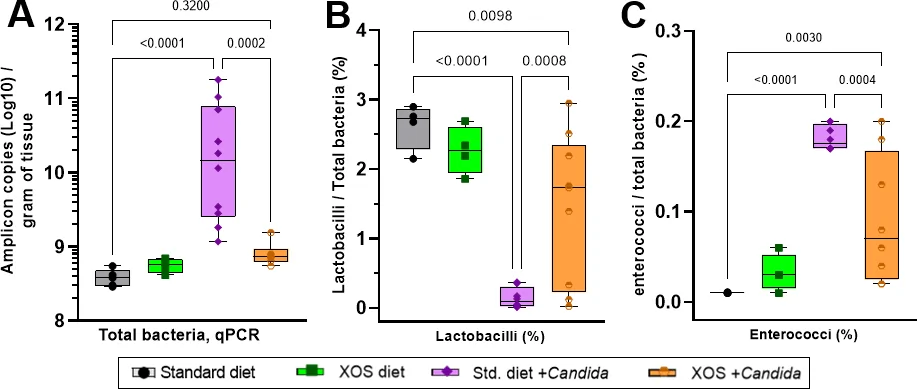
Effect of XOS diet on the oral bacteria microbiome of *Candida*-infected mice. (**A**) Total bacterial biomass by qPCR (16S gene amplicons); (**B**) qPCR of lactobacilli or (**C**) enterococci as a ratio of total bacterial biomass. One-way ANOVA with uncorrected Dunn’s test.

We previously reported that *C. albicans* infection induces dysbiotic changes in the murine mucosal bacterial microbiome, the most prominent being an increase in the total bacterial biomass and an increase in the relative abundance and biomass of enterococci (5). Therefore, we tested the effect of the XOS diet on these infection outcomes. As expected, total oral bacteria biomass was significantly increased in *Candida*-infected mice under the standard diet, compared to uninfected mice (Fig. 8A). In contrast, under the XOS diet, *Candida* infection did not cause an increase in the total bacterial biomass above control uninfected levels (Fig. 8A). We next evaluated the effect of XOS on lactobacilli, which are antagonistic to *Candida* (13, 15), and enterococci, which promote virulence in the mouse OPC model (13). In mice on the standard diet, *Candida* infection was associated with significantly reduced lactobacilli (Fig. 8B) and significantly increased enterococci (Fig. 8C). Importantly, the XOS diet prevented these infection-induced changes since the relative abundance of *Lactobacillus* and *Enterococcus* in the XOS diet was closer to that in the uninfected mice on a standard diet than in the *Candida*-infected mice on a standard diet. In summary, these findings show that XOS is associated with a protective effect against *Candida*-induced oral bacterial dysbiosis.

## DISCUSSION

*Lactobacillus* spp. represent the most abundant bacterial communities in the murine oral microbiome as we [(13), and this work] and others (21) have reported. An unexpected finding in this work was the reduction of resident oral lactobacilli in mice receiving an XOS diet. This was surprising because previous studies showed that XOS alone, or combined with *L. plantarum* inoculation, increased lactobacilli in the murine gut (17 – 19, 22). These studies were performed in different host genetic, nonimmunosuppressed backgrounds, and focused exclusively on the gut, which may explain the discrepancy with our findings. Site specificity for the effects of the XOS diet was reported in a study that compared the effect of XOS in the stomach versus the gut of the same mammalian host and found a significant increase in lactobacilli only in the gut (19), most likely related to different species colonizing different sites.

In untreated mice, 10 OTUs encompassed >80% of the oral taxa, as assessed by 16S rRNA gene V4 amplicon sequencing. Our results agree with another report of the oral bacterial microbiome composition in C57BL/6 mice from the same vendor, using a similar high-throughput sequencing approach (21). Our study shows that the core oral microbiome taxa of these mice include *Lactobacillus*, *Bifidobacterium*, *Sphingomonas*, *Streptococcus*, and certain unclassified bacteria. We found that a 3-week XOS diet induced changes in the murine oral bacterial composition and altered the structure of oral communities although it did not alter the total bacterial biomass. XOS was associated with a significant increase in the relative abundance of *Bifidobacterium*, which replaced lactobacilli as the most dominant taxon in the oral cavity, consistent with studies in the gut literature (19, 22). This is relevant because bifidobacteria have been associated with health benefits as probiotics, and some species have anticandidal activity (12, 23, 24).

In *Candida*-infected mice, XOS significantly attenuated the *Candida*-induced oral lesions and associated bacterial dysbiosis. Because XOS-induced changes in the oral microbiome were not maintained after discontinuing this diet (Fig. 5C, XOS→PBS group), it is reasonable to assume that *Candida* virulence would not be attenuated if the diet was discontinued during infection. The exact mechanisms associated with XOS-mediated attenuation of OPC remain to be explored, but these are more likely related to changes in the oral microbiota and *Candida* virulence rather than with host responses since all mice on this diet were immunosuppressed. However, testing this diet in non-immunosuppressed host models would be needed to rule this out. A reduction in oral lactobacilli in mice with *Candida* infection has been previously reported (13). In this study, we observed that the XOS diet enriched the oral lactobacilli populations in infected mice and reversed the infection-associated reduction in these microbial communities. Lactobacilli have a well-established protective role in intestinal and vaginal candidiasis (25 – 28). These organisms can interact with the host to stimulate antifungal responses such as positive regulation of tight junction proteins, or induction of epithelial antimicrobial peptides (29). A direct effect of the XOS-reconstituted oral lactobacilli on *Candida* virulence is also possible since we previously reported that strain MT4 displays both pH-dependent and pH-independent antibiofilm properties by secreting soluble metabolites with possible surfactin and/or glucanase activities (15). These mechanisms are currently being dissected further in our laboratory.

Since infection is associated with a reduction in lactobacilli, growth to repopulate this niche could be directly due to increased carbohydrate availability in the XOS-supplemented diet. However, XOS did not directly support the growth of oral lactobacilli recovered from swabs *in vitro*. Alternatively, the diet effects on lactobacilli in infected mice could be secondary to other changes in oral microbiota composition that promote metabolic cross-feeding in the presence of these carbohydrates. *L. johnsonii* is auxotrophic for several components of DNA and protein synthesis pathways and may have increased dependence on metabolic exchange with other species that hydrolyze XOS (30, 31).

A diet-focused study reported that coconut oil attenuated murine gastrointestinal candidiasis, which could be explained by the known direct anticandidal activity of this dietary supplement (32). In our study, XOS did not have a direct effect on *C. albicans* growth *in vitro*, or on indigenous fungal burdens *in vivo*. This finding, thus, further supports the possibility that attenuation of *Candida* virulence in the XOS diet OPC model is secondary to the increase in abundance of antagonistic lactobacilli and reduction in the abundance of potentially synergistic bacteria, such as enterococci. These findings are relevant because XOS are regarded as safe for human consumption by the FDA (USA) (33) and are approved by the European Union as prebiotics (34).

Another important finding was that after the prebiotic diet was discontinued, MT4 supplementation changed the oral microbiome composition to a structure that closely resembled the core structure of unperturbed communities. This finding suggests that, as a dominant species, *Lactobacillus johnsonii* may be a keystone organism that substantially influences the murine oral microbiome structure, and possibly function (35). Our findings are consistent with a report that gastric inoculation of mice with *L. jonhsonii* induces a significant change in the entire gut community phylogeny (36). In addition, another study found that, in a C57BL/6 mouse intestinal infection model, *L. johnsonii* restored the imbalance between aerobic and anaerobic bacteria and resulted in a significant reduction in inflammatory markers and an increase in chitinase-like protein-1 activity, which contributed to *Candida* clearance (37).

Oral microbiome relative abundance network analyses in C57BL/6 mice showed that in addition to being a dominant species, *L. johnsonii* is negatively correlated with oral bacterial taxa associated with increased *C. albicans* virulence, such as enterococci and streptococci (13). Because MT4 supplementation reduced the abundance of enterococci, we hypothesize that these previously reported negative correlations may be causal in nature (13). This is further supported by *in vitro* and *in vivo* studies showing antagonistic interactions of lactobacilli with these bacteria (13, 26, 38). Our previous studies also showed that the MT4 strain has certain genotypic and phenotypic attributes that may attenuate *C. albicans* virulence (13, 15). Additionally, *L. johnsonii* MT4 is closely related to *L. johnsonii* NCC 533 (La1) (15), a well-studied probiotic with multiple benefits to the human host (39, 40). Therefore, it is possible that this strain may provide a novel, well-targeted microbiome-based probiotic approach for therapeutic applications in the treatment or prevention of OPC, and further studies are needed in this direction.

## MATERIALS AND METHODS

### Strains and culture conditions

*L. johnsonii* strain MT4 was subcultured in MRS broth (Difco), anaerobically at 37°C for 24 h. Overnight cultures were washed with PBS, adjusted via spectrophotometry to OD_600_ = 0.2, and grown to a final OD_600_ = 1 prior to experiments. *C. albicans* SC5314 was subcultured in YPD broth, aerobically at 30°C on a shaker overnight prior to all experiments.

### Animals

Five- to 7-week-old C57BL/6 female mice from Jackson Laboratories were used in all experiments (animal protocol 102251-0323). Mice were acclimatized for 1–2 weeks before interventions. One day before the experiments, they were randomly sorted into the control and experimental groups, placed in corresponding cages, weighed, and orally swabbed to assess their initial oral microbial composition. *Research ethics*: All animal studies performed in this work were in compliance with the guide for the care and use of laboratory animals of the National Institutes of Health recommendations, the Animal Welfare Act federal regulations, and the University of Connecticut Institutional Animal Use and Care Committee (IACUC) guidelines. All protocols used in this study were approved by the UConn Health IACUC committee (IACUC protocol #102251-0323).

### XOS-supplemented diet

An XOS-enriched diet was provided daily, *ad libitum*, for 3 weeks. XOS (XOS-95 powder, Heagreen, China) was mixed with a standard powder diet (T.2918M.CS diet, Envigo, USA) in a 1:10 (wt/wt) ratio. Both diets showed a similar consistency. XOS was also provided daily in the drinking water as 10% (wt/vol) XOS solution. The standard diet with sterile drinking water was used as a control.

### Microbial growth in XOS-supplemented media

Cotton swabs from the oral cavity of naïve C57BL/6 mice were collected and vortexed in 500 µL of sterile PBS. Next, 100 µL of the bacterial suspension was transferred to 5 mL of standard MRS broth (2% dextrose) or MRS broth without dextrose supplemented with 10% XOS and incubated anaerobically at 37°C for 48 h. Microbial growth was measured spectrophotometrically (*λ* = 600 nm) every 60 min for the first 8 h and at *t* = 24 and 48 h. Two hundred microliters of bacterial suspension were collected, washed in PBS, and stored at −80°C for qPCR analysis. To further assess whether XOS has direct anticandidal activity, *C. albicans* was subcultured in an XOS-enriched YPD broth (2% and 10%, wt/vol), aerobically, at 30°C. Microbial growth was measured spectrophotometrically (*λ* = 600 nm) every 30 min, for 16 h.

### *L. johnsonii* MT4 oral inoculation in mice

In some experiments, MT4 was administered after cessation of the XOS diet in order to evaluate its role in the post-diet recovery of the oral bacterial communities. In these experiments, MT4 was orally inoculated (10^9^ bacterial cells in 50 µL of sterile water) in cortisone-immunosuppressed, anesthetized mice, twice in the course of a week, following cessation of the 3-week XOS diet (Fig. 1A).

### Oropharyngeal candidiasis model

To test the effect of the XOS diet on the severity of OPC, mice were infected with *C. albicans* during the third week of this diet, following an established protocol in our lab (5, 7, 13, 41) (Fig. 1B). One day prior to infection, mice were immunosuppressed with cortisone acetate (225 mg kg^−1^ body weight, injected subcutaneously). The next day, mice were anesthetized [ketamine: xylazine (90–100 and 10, mg kg^−1^) of body weight, respectively, via intramuscular injection] and infected subglossally with *Candida* (10^8^ yeast cells in cotton pellets). Cotton pellets with PBS only were orally administered under anesthesia to the control groups. The immunosuppression/infection step was performed twice over the course of 5 days. Mice were sacrificed on day 5, and tongues were harvested for microbial analyses and macroscopic tissue evaluation. Mice kept on the standard diet during the same period served as controls.

### Data/sample collection and analyses

#### Body weight

Changes in body weight were monitored as a sign of animal morbidity and expressed as a percentage of initial weight.

#### Diet consumption

Consumption of food (grams) and water (mL) was measured daily, as a sign of acceptance of the XOS diet and distress associated with infection.

#### Assessment of tongue lesions

Tongues were removed aseptically at necropsy, photographed, and images were saved as jpg files. Images were subsequently analyzed using the NIH Image J software (http://rsb.info.nih.gov/ij), and data were expressed as percent surface area covered by biofilm (total surface area of white lesions/entire tongue dorsal surface area). Previous studies have confirmed that this method of assessment correlates well with histologic evidence of tissue damage (7, 41).

#### CFU quantification

Excised tongues were weighed and homogenized. Homogenates were serially diluted and plated for viable counts as follows: Rogosa agar for lactobacilli, CATC (Citrate Azide Tween Carbonate) base agar for enterococci (42), and Brain Heart Infusion agar for total bacteria. For bacterial recoveries, plates were incubated anaerobically at 37°C for 3–5 days. For *Candida* quantification, homogenates were plated in Sabouraud Dextrose agar supplemented with chloramphenicol (10 µg/mL) and incubated aerobically at 37°C for 48–72 h.

#### DNA extraction and microbial qPCR

Samples were incubated overnight using a custom lysis buffer optimized for oral microbiome characterization in murine models (43) and then were homogenized using zirconia beads (Ambion, USA) in a Fastprep-24 (MP Biomedicals, USA) to disrupt the bacterial cells. Tongues were processed the next day using the Qiagen DNA Blood and Tissue mini kit. DNA from fecal pellets was isolated using the QIA DNA Stool kit, following the manufacturer’s recommendations. DNA quantity and quality were evaluated using a NanoDrop. Microbial biomass was quantified using 16S rRNA gene copy numbers for total bacteria and bifidobacteria [genus-level (44)], 16S/23S rRNA intergenic spacer region for lactobacilli [genus-level (45)], the *tuf* gene copies for enterococci [genus-level (46)], the ONDMJEFH_01749 hypothetical protein gene copies for *L. johnsonii* MT4 (strain-specific, this work), and the 18S/28S rRNA intergenic region for *Candida* (47). Primers and amplification conditions are described in Table S2.

#### Oral microbiome characterization

DNA, extracted from tongues, was quantified using the Quant-iT PicoGreen kit (Invitrogen). 16S rRNA genes were amplified in triplicate using 30 ng of extracted DNA as template. The V4 region was amplified using 515F and 806R primers with Illumina adapters and bar codes on the 3′ end (48). PCR products were pooled for quantification and visualization using the QIAxcel DNA Fast Analysis kit (Qiagen). Pooled PCR products were then processed using the Mag-Bind RxnPure Plus kit (Omega Bio-tek) according to the manufacturer’s protocol, to include only sequences between 250 and 400 bp. The cleaned pool was sequenced on the MiSeq using a v2 2 × 250 bp kit (Illumina). Sequences were processed following a standard pipeline (49) and classified using Mothur’s version of the Ribosomal Database Project classifier (Mothur v1.39.5) (50).

For OTU analyses, sequences were clustered using a 97% similarity cutoff and classified up to the genus level based on the consensus taxonomy. Alpha and beta diversity statistics were calculated by subsampling 1,000 reads per sample. Alpha diversity was measured via the Shannon diversity index and OTU richness in each sample. The relative abundance of OTUs of the main genera was plotted by using stack bar graphs, including the 10 most abundant OTUs, using the untreated groups as a reference. Non-metric multidimensional scaling (NMDS) ordination was used to observe community clusters associated with Bray-Curtis dissimilarity calculations. NMDS plots were used to survey bacterial OTU heterogeneity relative to that in each treatment, and permutational multivariate analyses of variance comparisons of the Bray-Curtis dissimilarity distances were performed. All analyses were conducted in R version 4.0.3 (http://www.r-project .org). Oral microbiota variability between mouse batches and providers has been reported by others (21, 51, 52), so each intervention was tested in the same batch of mice.

## Data Availability

All sequence data sets have been deposited to the NCBI database. BioProject under accession number PRJNA984278 contains the following BioSamples and corresponding raw SRAs for each experimental condition: standard diet (SAMN35755168), XOS (SAMN35715379), XOS->MT4 (SAMN35758582), XOS->PBS (SAMN35758760).

## SUPPLEMENTAL MATERIAL

Supplemental materials spectrum.01734-23-s0001.docx Supplemental figures and tables.

ASM does not own the copyrights to Supplemental Material that may be linked to, or accessed through, an article. The authors have granted ASM a non-exclusive, world-wide license to publish the Supplemental Material files. Please contact the corresponding author directly for reuse.
