# Supplementary material for: A prebiotic diet modulates the oral microbiome composition and results in the attenuation of oropharyngeal candidiasis in mice
Source: Microbiol Spectr. 2023 Sep 6;11(5):e01734-23. doi: 10.1128/spectrum.01734-23 (PMC10580959; doi:10.1128/spectrum.01734-23)
Supplement: Supplemental materials — Supplemental figures and tables. [file spectrum.01734-23-s0001.docx]

**SUPPLEMENTARY MATERIALS**

**A prebiotic diet modulates the oral microbiome composition and results in the attenuation of oropharyngeal candidiasis in mice.**

Roberto Vazquez-Munoz, Angela Thompson, Takanori Sobue and Anna Dongari-Bagtzoglou

Supplementary Figures

**Figure S1**


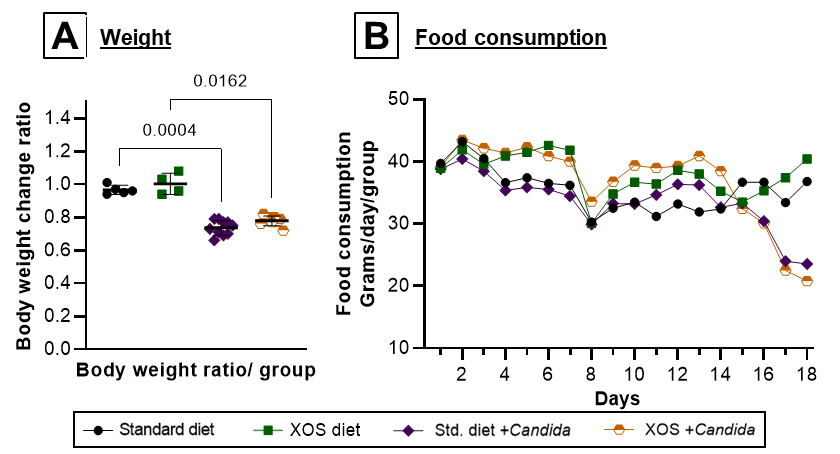


**Sup. Fig. S1**. Body weight and food consumption. (A) Body weight expressed as ratio of initial/final weight in grams (B) Food consumption over time (expressed in grams per day/group). One-Way ANOVA with the Uncorrected Dunn’s Test.

**Figure S2.**


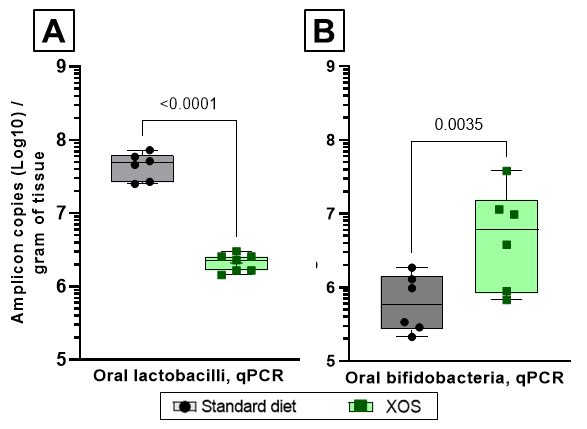


**Sup. Fig. S2**. Effect of dietary XOS on oral lactobacilli (A), and bifidobacteria (B), by genus-specific qPCR. Unpaired t-test.

**Figure S3.**


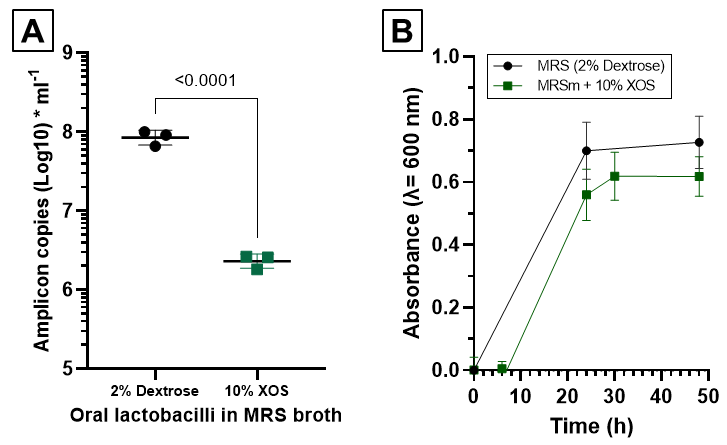


**Sup. Fig. S3**. Effect of XOS on microbial growth *in vitro*. *Lactobacillus* biomass in MRS supplemented with 10% XOS or 2% dextrose after 24 hours of incubation (A). Bacterial growth over time in MRS broth supplemented as above (B). Unpaired t-test.

**Figure S4.**


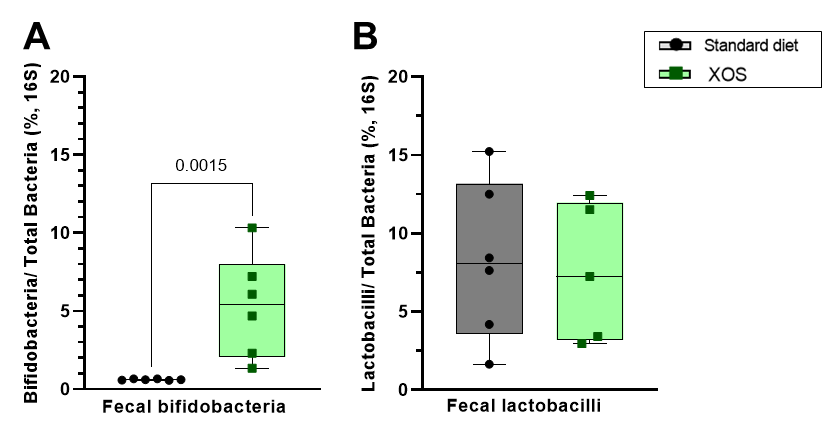


**Sup. Fig. S4**. Effect of dietary XOS in fecal biomass of bifidobacteria (A) and lactobacilli by genus specific qPCR (B). Unpaired t-test.

**Figure S5.**


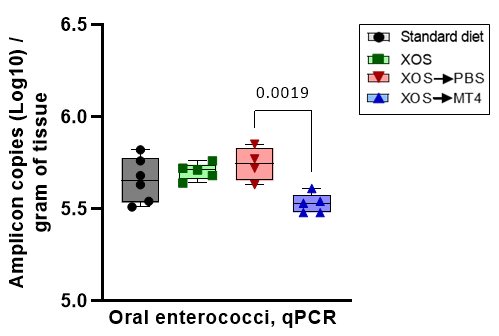


**Sup. Fig. S5**. Enterococcal biomass in the four groups as assessed by genus-specific qPCR.

**Figure S6.**


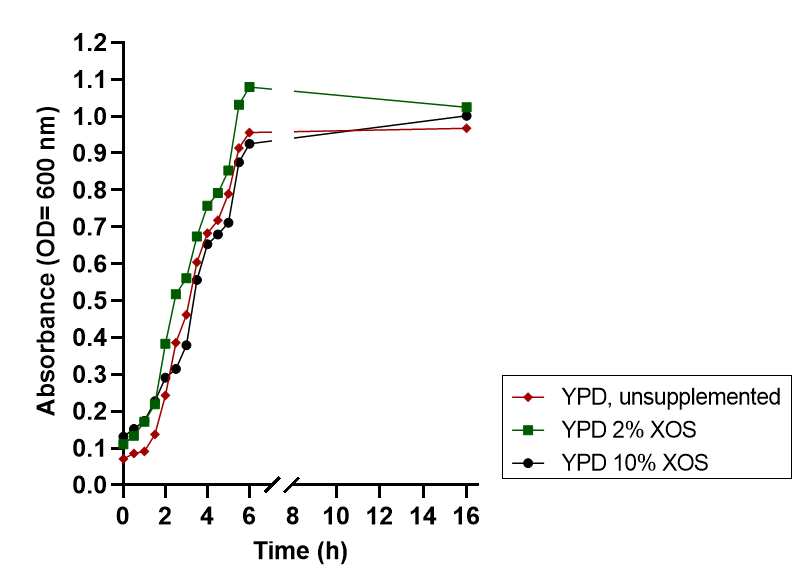


**Sup. Fig. S6**. Effect of XOS on *C. albicans* growth in vitro. *C. albicans* blastospores were grown in YPD broth enriched with XOS (2% or 10%, w/v). XOS supplementation had no effect on *Candida* growth.

Supplementary tables

**Table S1. Most common OTUs in the oral cavity of mice in each dietary group**.

| **Table S1**. Most common OTUs in the oral cavity of mice in each dietary group. | | | |
| --- | --- | --- | --- |
| **Standard diet** | **XOS** | **XOS → PBS** | **XOS → MT4** |
| ***Lactobacillus* (28.7% ±6.5)**  *Sphingomonas* (20% ±9.1)  *Streptococcus* (9.1% ±4.3)  ***Bifidobacterium* (9.1% ±2.7)**  OTU#009* (8.5% ±3.5)  *Staphylococcus* (5% ±3.5)  *Enterococcus* (0.6% ±0.6)  OTU#013* (0.1% ±0.1)  OTU#005* (0% ± 0)  OTU#011* (0% ±0)  Other taxa (18.8%) | ***Bifidobacterium* (40.8% ±4.4)**  ***Lactobacillus* (15.6% ±7.3)**  OTU#011* (5.9% ±3.7)  OTU#009* (5.4% ±0.7)  OTU#013* (4.4% ±3.4)  *Sphingomonas* (3.8% ±1.4)  *Streptococcus* (3.7% ±2.8)  *Staphylococcus* (0.6% ±0.7)  OTU#005* (0.5% ±0.3)  *Enterococcus* (0.2% ±0.2)  Other taxa (19.3%) | ***Lactobacillus* (27.5% ±14.8)**  *Sphingomonas* (7.5% ±1.3)  *Staphylococcus* (6.5% ±3.3)  *Streptococcus* (3.2% ±0.2)  OTU#005* (2.8% ±1.8)  *Enterococcus* (1.6 % ±1.5)  OTU#013* (1.1% ±1.3)  ***Bifidobacterium* (0.8% ±0.4)**  OTU#009* (0.5% ±0.4)  OTU#011* (0.8% ±0.7)  Other taxa (47.7%) | ***Lactobacillus* (34.5% ±10.9)**  OTU#005* (20.8% ±14.3)  *Sphingomonas* (17.9% ±13.7)  *Streptococcus* (8.3% ±5.9)  OTU#009* (4.6% ±1)  ***Bifidobacterium* (4% ±1.6)**  OTU#011* (0.4% ±0.5)  OTU#013* (0.2% ±0.2)  *Enterococcus* (0%)  *Staphylococcus* (0%)  Other taxa (9.4%) |
| * Unclassified bacteria  Bacteria are organized by decreasing average relative abundance in each group (n= 5 mice/group).  Putative probiotic bacteria are in bold, whereas OPC-associated bacteria are underlined. | | | |

**Table S2.** Primers

| **Table S2.** Primers used in this work. | | | | |
| --- | --- | --- | --- | --- |
| **Primer set** | | **Sequence 5’ - 3’** | **Product size (bp)** | **Reference** |
| *Lactobacillus*, genus-level | R16-1 | CTTGTACACACCGCCCGTCA | 209 - 250 | Dubernet, et al 2002 ^43^ |
|  | LbLMA1-rev | CTCAAAACTAAACAAAGTTTC |  |  |
| Total bacteria,  16S rRNA | 16S | GATACATAGCCGACCTGAG | 98 | Periasamy, et al 2009* ^51^ |
|  | 16S | TCCATTGCCGAAGATTCC |  |  |
| Bifidobacteria, genus-level | g-Bifid-F | CTCCTGGAAACGGGTGG | 549 - 563 | Matsuki, et al 2002 ^42^ |
|  | g-Bifid-R | GGTGTTCTTCCCGATATCTACA |  |  |
| Bifidobacterium pseudolongum, species-level | IDB41F | CCCTTTTTCCGGGTCCTGT | 471 | Kwon, et al 2005^52^ |
|  | IDBC1R | ATCCGAACTGAGACCGGTT |  |  |
| *Enterococcus*, genus-level | ENT1 (Fw) | TACTGACAAACCATTCATGATG | 112 | Ke, et al 1999 ^44^ |
|  | ENT2 (Rv) | AACTTCGTCACCAACGCGAAC |  |  |
| Total fungi,  5.8S-28S region | FP 5.8S | GTGAATCATCGARTCTTTGAAC | 242-269 | Khot, et al 2009 ^45^ |
|  | RP 28S-1 | TATGCTTAAGTTCAGCGGGTA |  |  |

PCR conditions: Thermal cycler (BioRad). The total reaction mixture is 20 μL per sample: 10 μL iTaq Universal SYBR® Green Supermix (Bio-Rad cat. #1725121), 1 μL of each 10 μM Forward and Reverse primers, 3 μL DNase-Free Water, and 5 μL of DNA template.

For total bacteria quantification, the 16S primers were first reported as species-specific for *Streptococcus oralis*; however, *in* *silico* analyses (NLM’s Primer Blast Tool, https://www.ncbi.nlm.nih.gov/tools/primer-blast/) and qPCR experimental data confirms these primers amplify multiple species from more than 10 bacterial genera, including all our bacteria of interest.
